# URBANIZATION, FALLS, AND FEAR OF FALLING IN COMMUNITY-DWELLING OLDER ADULTS IN THAILAND

**DOI:** 10.1093/geroni/igad104.0785

**Published:** 2023-12-21

**Authors:** Ladda Thiamwong, Rui Xie, Sirinart Tongsiri, Boon Peng Ng, Joon-Hyuk Park, Jaroonrat Rodniam, Wenjun Li, Elizabeth Eckstrom

**Affiliations:** University of Central Florida, Orlando, Florida, United States; University of Central Florida, Orlando, Florida, United States; Mahasarakham University, Mahasarakham, Maha Sarakham, Thailand; University of Central Florida, Orlando, Florida, United States; University of Central Florida, Orlando, Florida, United States; Panyapiwat Institute of Management, Sattahip, Chon Buri, Thailand; University of Massachusetts Lowell, Lowell, Massachusetts, United States; Oregon Health & Science University, Portland, Oregon, United States

## Abstract

Nearly half of the Thai population lives in urban areas without retirement pensions. Urbanization may increase physical inactivity, falls, and other non-communicable diseases. Little is known about the urban-rural differences in falls and fear of falling among community-dwelling older in Thailand. We surveyed a random sample of 433 persons who were: i) aged 60 years and older; and ii) had no cognitive impairment (Chula Mental Test ≥ 16/36). The participants had the mean age was 70.40 (± 7.53) years, included 62.9 % female, 22% having poor balance, 85% having limits in doing vigorous physical activity, 71% having limits in performing moderate physical activity, and 63.5% having pain interfere with normal activities. About half had financial problems sometimes, and 68 % reported living with a spouse and children. 30% had hypertension and took antihypertensive medication. 16% felt tired most of the time, and 11% felt nervous all the time. We found that urban older adults had a higher fall rate than rural older adults (52.8% vs. 40.4%, p =.034). Urban older adults also had a higher level of fear of falling than rural older adults (43.9% vs. 28.4%, p = .000016). Additionally, urban older adults had significantly worse general health than rural older adults (p =.0046). There were no significant differences in balance performance and fall self-efficacy scores. It is important to recognize and identify fall risk factors among urban older adults and provide access to systematic community-based fall prevention programs incorporating screening and tailored interventions based on those risk factors.

